# Aortic Aneurism—Report of a Case Measuring Five by Eight Inches in Diameter and Weighing Five Pounds

**Published:** 1882-07

**Authors:** Chas. C. F. Gay

**Affiliations:** Buffalo, N. Y.


### Aortic Aneurism—Report of a Case Measuring Five by Eight Inches in Diameter and Weighing Five Pounds.

By Chas. C. F. Gay, M. D., Buffalo, N. Y.

W. G. B., aet. 61, for many years actively engaged as principal partner in a large Dry Goods House of this city, died on October 16th, 1881, of aortic aneurism of about four years' duration.

### HISTORY AND SYMPTOMS.

On January 14th, 1877, Mr. B. was suddenly taken ill at night. His symptoms were alarming and of a character to give rise to the apprehension that he had fatal cardiac disease.

The pulsations of the heart were feeble; he was faint; his extremities cold, and there was some pain that resembled that of angina pectoris. I was called to him twice the same night.

He was detained from business not more than a day or two and never had another illness of the same nature. Sometime subsequent to this attack he had severe pain in his foot—he may have had it before—which was believed to be rheumatism. He thought this pain of the foot of the same character which he afterwards had in his shoulder; he had repeated attacks of it up to a short time prior to his death. With this exception his previous health had been good; he had never received injury or had any constitutional or inherited ailment. One sister had a tumor of the breast, another had been an invalid many years.

About three years prior to his death he began to have pain in his shoulders and back part of the neck, extending up to the back part of the head. This was supposed to be also rheumatic, and was at times so severe as to destroy his rest at night, but which yielded not the least to rheumatic remedies.

Nothing but respite from the cares of his large business had any remedial effect. Three or four weeks' rest and travel would restore him to apparent health. Three or four of these seizures occurred annually. On July 11th, 1880, I observed unusual pulsation at the superior border, and at the left of the median line of the sternum. There was no tumor visible at this time—fifteen months previous to death—and the pulsations had been unnoticed by the patient.

At length a small tumor presented itself, in which there was pulsation, but never at any time during the progress of its growth was there bruit. The tumor enlarged rapidly until, shortly before death, it measured eight inches in its long and five inches in its short diameter. It extended from the right side of the sternum across it, reaching well up the neck in front and terminating midway between the left side the neck and outer aspect of the shoulder.

On his return from an eastern visit early in the month of May he coughed almost incessantly for three or four weeks in consequence, he thought, of a cold contracted during his absence. Finally his cough entirely left him. The cough was undoubtedly partly caused by the cold, but chiefly I think by pressure of the tumor upon the bronchus. The immediate cause of death was suffocation.

Pressure during the last few days of the patient's life upon the respiratory organs and œsophagus, caused dyspnœa and dysphagia—the breathing resembling an asthmatic attack.

TREATMENT

consisted of occasional administration of Pot. Iod. in doses varying from grs. v to xxv. Tr. veratrum veride, with digitalis, controlled the heart's action and maintained the pulse at 60 beats per minute. Inf. prunus virg. and, later in the case, anodynes in full doses. Recumbent posture and rest in bed. Straps of Ad. plaster were applied over the tumor, the walls of which became so thin and the pulsations, at times, so violent that rupture was feared.

AUTOPSY, made by Dr. B. Bartow in the presence of many members of the profession, confirmed the diagnosis, revealing an aneurism, which had its origin at the arch of the aorta and which, with the heart, weighed *five and a half pounds.* It was laminated and contained a blood clot. The upper section of the sternum was carried away by the process of erosion, caused by pressure upon it.

On account of the destruction of a portion of the sternum the sternal extremity of the left clavicle and two of the ribs had no attachment. The heart was normal in size and structure. After deducting the weight of *the normal heart* the weight of the aortic tumor is fully *five pounds.* It seems quite incredible that a tumor should grow to this size, push its way through flesh, cartilage and bone without rupture. Aortic tumors of larger dimensions may have been reported, but this exceeds in size any of which I have knowledge.

